# Tracking drug-resistant Streptococcus pneumoniae in Oregon: an alternative surveillance method.

**DOI:** 10.3201/eid0505.990510

**Published:** 1999

**Authors:** A E Chin, K Hedberg, P R Cieslak, M Cassidy, K R Stefonek, D W Fleming

**Affiliations:** Oregon Health Division, Portland, Oregon, USA. gchin@javanet.com

## Abstract

With the emergence of drug-resistant Streptococcus pneumoniae, community-specific antimicrobial susceptibility patterns have become valuable determinants of empiric therapy for S. pneumoniae infections. Traditionally, these patterns are tracked by active surveillance for invasive disease, collection of isolates, and centralized susceptibility testing. We investigated whether a simpler and less expensive method aggregating existing hospital antibiograms--could provide community-specific antimicrobial susceptibility data. We compared 1996 active surveillance data with antibiogram data from hospital laboratories in Portland, Oregon. Of the 178 S. pneumoniae active surveillance isolates, 153 (86% [95% confidence interval (CI) = 80% to 91%]) were susceptible to penicillin. Of the 1,092 aggregated isolates used by hospitals to generate antibiograms, 921 (84% [95% CI = 82%-87%]) were susceptible to penicillin. With the exception of one hospital's erythromycin susceptibility results, hospital-specific S. pneumoniae susceptibilities to penicillin, cefotaxime, trimethoprim-sulfamethoxazole, and erythromycin from the two methods were statistically comparable. Although yielding fewer data than active surveillance, antibiograms provided accurate, community-specific drug-resistant S. pneumoniae data in Oregon.


### Tracking Drug-Resistant *Streptococcus pneumoniae* in Oregon: An Alternative Surveillance Method

**Arthur E. Chin,*† Katrina Hedberg,* Paul R. Cieslak,* Maureen Cassidy,* Karen R. Stefonek,* and David W. Fleming***
*Oregon Health Division, Portland, Oregon, USA; and †Centers for Disease Control and Prevention, Atlanta, Georgia, USA

With the emergence of drug-resistant *Streptococcus pneumoniae,* community-specific antimicrobial susceptibility patterns have become valuable determinants of empiric therapy for *S. pneumoniae* infections. Traditionally, these patterns are tracked by active surveillance for invasive disease, collection of isolates, and centralized susceptibility testing. We investigated whether a simpler and less expensive method—aggregating existing hospital antibiograms—could provide community-specific antimicrobial susceptibility data. We compared 1996 active surveillance data with antibiogram data from hospital laboratories in Portland, Oregon. Of the 178 *S. pneumoniae* active surveillance isolates, 153 (86% [95% confidence interval (CI) = 80% to 91%]) were susceptible to penicillin. Of the 1,092 aggregated isolates used by hospitals to generate antibiograms, 921 (84% [95% CI = 82%-87%]) were susceptible to penicillin. With the exception of one hospital's erythromycin susceptibility results, hospital-specific *S. pneumoniae* susceptibilities to penicillin, cefotaxime, trimethoprim-sulfamethoxazole, and erythromycin from the two methods were statistically comparable. Although yielding fewer data than active surveillance, antibiograms provided accurate, community-specific drug-resistant *S. pneumoniae* data in Oregon.

*Streptococcus pneumoniae* infections are a major cause of illness and death worldwide. Penicillin-resistant *S. pneumoniae* were first described in 1967 (1). Since then, the proportions of isolates resistant to penicillin and other antimicrobial agents have increased worldwide (2-5). In the United States, the combined percentage of *S. pneumoniae* isolates with either intermediate (MIC = 0.1-1.0 μg/ml) or high (MIC 2.0 μg/ml) levels of penicillin resistance is higher than 60% in some areas (5). Strains with multidrug resistance to penicillins, macrolides, sulfonamides, and third-generation cephalosporins have been well documented (3,5-9). Despite the increasing proportion of drug-resistant *S. pneumoniae* and the importance of knowing the drug resistance status in determining empiric therapy, community-specific surveillance for drug-resistant *S. pneumoniae* is limited and its proportion is unknown in many areas (10,11). Active surveillance for invasive *S. pneumoniae* disease includes collection of isolates, centralized susceptibility testing, and collection of patient data (4).

Although such a resource-intensive system for providing community-specific and case-specific *S. pneumoniae* data is beyond the means of most local and many state health agencies, hospital-specific data already exist in many areas. Many hospital laboratories perform antimicrobial susceptibility testing on *S. pneumoniae* isolates from sterile and nonsterile sites, and results are often tabulated for local clinicians in a summary table called an antibiogram. Antibiogram data represent invasive and noninvasive *S. pneumoniae* disease isolates collected from normally sterile and nonsterile sites; may include multiple isolates from the same patient; and are based on hospital laboratory antimicrobial susceptibility testing, a process that may differ between laboratories. In contrast, *S. pneumoniae* active surveillance data are limited to invasive disease isolates collected

Address for correspondence: Arthur E. Chin, 63 Indian Mountain Road, Lakeville, CT 06039, USA; fax: 860-364-4427; e-mail: gchin@javanet.com <mailto:gchin@javanet.com.

from normally sterile sites, specifically exclude duplicate isolates collected from the same patient, and are based on a centralized and standardized susceptibility testing protocol.

We examined preexisting antibiogram data to assess if they could provide local health agencies with an accurate, inexpensive means of estimating the community-specific proportion of drug-resistant *S. pneumoniae*. The Oregon Health Division performs active surveillance for drug-resistant *S. pneumoniae* through a cooperative agreement with the Centers for Disease Control and Prevention's Emerging Infections Program. We conducted a cross-sectional survey of the 12 hospital laboratories that serve the Portland Tri-County area (Multnomah, Washington, and Clackamas counties, population 1.2 million) and compared 1996 Portland *S. pneumoniae* susceptibility results and costs of the aggregated antibiogram surveillance system with the *S. pneumoniae* susceptibility results and costs of our active surveillance system. We determined the community-specific proportion of *S. pneumoniae* susceptible to penicillin and performed a limited analysis of hospital-specific susceptibilities to cefotaxime, trimethoprim-sulfamethoxazole, and erythromycin.

### Methods

### Active Surveillance

#### Case Definition

Our goal was to determine the proportion of drug-resistant isolates among all *S. pneumoniae* isolates collected by the active surveillance system in 1996. Therefore, an active surveillance case was defined as an *S. pneumoniae* isolate from a normally sterile site collected from a Portland Tri-County resident in 1996 and analyzed at a Portland Tri-County hospital microbiology laboratory.

#### Surveillance Protocol

All Portland-area hospital microbiology laboratories were asked to send all *S. pneumoniae* sterile-site isolates from both inpatients and outpatients to the Oregon State Public Health Laboratory. Health Department staff regularly contacted each laboratory to assess interim isolate recovery rates and to encourage ongoing participation in the surveillance system and (twice a year) performed on-site laboratory audits to compare the number of patients with invasive *S. pneumoniae* infections with the number of isolates submitted to the state laboratory.

To avoid duplication, only one isolate from each patient was sent to the reference laboratory, even if multiple isolates were obtained from the same person. Isolates were sent twice a year from the Oregon State Public Health Laboratory to a national reference laboratory for antimicrobial susceptibility testing by National Committee for Clinical Laboratory Standards broth microdilution protocols (12). *S. pneumoniae* antimicrobial-susceptibility percentages for Portland were calculated from the national reference laboratory results. Invasive cases did not have reference laboratory susceptibility testing if the hospital laboratory did not forward the isolate to the Oregon State Public Health Laboratory or if the isolates received by the Oregon or the reference laboratory were not viable.

#### Cost Calculations

Annual costs for this surveillance system included direct and indirect health department staff costs and the expense of isolate storage, processing, and transport incurred by the Oregon State Public Health Laboratory and the national reference laboratory. Hospital laboratory isolate testing, which would have been performed regardless of our request for surveillance data, were not included in these calculations. Time calculations included laboratory audits, patient chart reviews, data entry and analysis, coordination of isolate movement, and communication among hospital laboratories, the health department, the state public health laboratory and the reference laboratory.

### Antibiogram Surveillance

#### Case Definition

An antibiogram case was defined as any *S. pneumoniae* isolate identified in 1996 by a Portland Tri-County hospital microbiology laboratory that was tabulated on the respective 1996 *S. pneumoniae* antibiogram. Specimens were submitted from inpatients and outpatients and from sterile and nonsterile sites.

#### Surveillance Protocol

We requested antibiograms from all 12 Portland Tri-County hospital laboratories.

Antibiogram data were aggregated to produce antimicrobial susceptibility percentages for the Portland area. All susceptibility testing was performed at individual hospital laboratories. We did not routinely survey laboratory techniques or reporting criteria, nor did the Oregon State Public Health Laboratory perform confirmation susceptibility testing of any hospital isolates.

### Cost Calculations

The cost of the antibiogram method included direct and indirect health department staff expenses but excluded the cost of hospital laboratory isolate testing, a process performed regardless of our surveillance requests. Time calculations included staff time spent requesting antibiograms and performing data entry and analysis.

### Statistical Methods

The Mantel-Haenzel chi-square and Fisher's exact tests were used to compare the proportions of susceptible *S. pneumoniae* isolates determined by the two surveillance methods. P values ≤0.05 were considered statistically significant. Statistical calculations were performed by using Epi-Info (Epi-Info version 6.04b; Centers for Disease Control and Prevention, Atlanta, GA).

### Findings

### Penicillin

Of the 12 Portland-area hospital laboratories participating in the active surveillance system, 10 (83%) submitted isolates to our active surveillance system in 1996. One hospital (A) had no sterile-site isolates in 1996. A second hospital (F) had two sterile-site isolates but did not submit them to the state laboratory. Of 266 invasive *S. pneumoniae* infections identified by health department staff through audits, 178 (67%) *S. pneumoniae* isolates were tested by the reference laboratory. Of the 88 identified cases that were not analyzed by the reference laboratory, in 81 cases the hospital did not submit an isolate to the state laboratory, and in 7 the isolate submitted was not viable. The number of isolates collected from each hospital was 2 to 59 (Table 1). The mean and median numbers of active surveillance isolates collected per hospital were 18 and 9.5, respectively. Of the 178 isolates tested, 153 (86% [95% CI = 80% to 91%]) were susceptible to penicillin (MIC  0.06 µg/ml).

Table 1. *Streptococcus pneumoniae* penicillin susceptibility as determined by two surveillance methods, Portland, Oregon, 1996

| | Active surveillance | | | Antiobiograms | | |
|---|---|---|---|---|---|---|
| | No. | Susceptible | | No. | Susceptible | |
| Hospital | isolates | N | % | isolates | N | % |
| A | [a] | | | 20 | 17 | 85 |
| B | 34 | 29 | 85 | 134 | 112 | 84 |
| C | 59 | 52 | 88 | 274 | 227 | 83 |
| D | 6 | 4 | 67 | 120 | 89 | 74 |
| E | 12 | 11 | 92 | 41 | 34 | 83 |
| F | [a] | | | 61 | 58 | 95 |
| G | 33 | 28 | 85 | 110 | 100 | 91 |
| H | 11 | 10 | 91 | 161 | 137 | 85 |
| I | 8 | 6 | 75 | 64 | 56 | 88 |
| J | 7 | 6 | 86 | 107 | 91 | 85 |
| K | 6 | 5 | 83 | [b] | | |
| L | 2 | 2 | 100 | [b] | | |
| Total | 178 | 153 | 86 | 1,092 | 921 | 84 |

[a]No isolates submitted to the Oregon Public Health Laboratory.
[b]Antibiogram data not available from hospital.

Penicillin antibiogram data were collected from 10 (83%) of 12 Portland-area hospitals (Table 1). Eight of the 10 hospitals listed only the proportion of susceptible *S. pneumoniae* isolates on their antibiogram and did not specify the number of intermediate- or high-resistance isolates. Of the aggregated 1,092 *S. pneumoniae* isolates used by Portland-area hospitals to generate penicillin antibiogram data, 921 (84% [95% CI = 82% to 86%]) were listed as susceptible to penicillin.

The proportion of penicillin-susceptible isolates at each hospital was 67% to 100% by the active surveillance method and 74% to 95% by antibiogram data (Figure). The median hospital-specific difference between the two methods

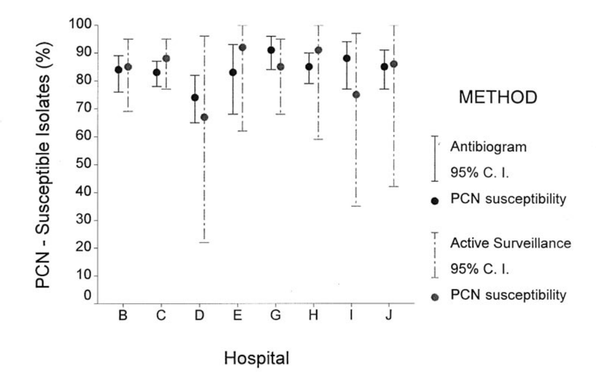

Figure. Hospital-specific *Streptococcus pneumoniae* penicillin-susceptibility determined by antibiogram and active surveillance, Portland, Oregon, 1996.

was 6%. In no instance did hospital-specific penicillin-susceptibility estimates from the two methods differ statistically (p >0.05). We found no statistical difference between the overall *S. pneumoniae* penicillin-susceptibility proportion determined by active surveillance and by the antibiogram method (p >0.05).

### Other Antibiotics

We compared active surveillance and antibiogram *S. pneumoniae* susceptibilities to cefotaxime, trimethoprim-sulfamethoxazole, and erythromycin (Table 2). Of the 178 isolates collected and tested through the active surveillance system, 165 (93% [95% CI = 88% to 96%]) were susceptible to cefotaxime (MIC 0.50 µg/ml), 141 (79% [95% CI = 73% to 85%]) were susceptible to trimethoprim (MIC 0.50 µg/ml)-sulfamethoxazole (MIC 9.50 µg/ml), and 169 (95% [95% CI = 91% to 98%]) were susceptible to erythromycin (MIC 0.50 µg/ml).

In hospitals where antibiogram data were available for cefotaxime, trimethoprim-sulfamethoxazole, and erythromycin, 539 (94%) of 575 aggregated isolates (95% CI = 91% to 95%) were susceptible to cefotaxime, 251 (84%) of 300 isolates (95% CI = 79% to 88%) were susceptible to trimethoprim-sulfamethoxazole, and 649 (86%) of 751 isolates (95% CI = 84% to 89%) were susceptible to erythromycin. Hospital-specific antibiogram and active surveillance data from four institutions were available for direct comparison for cefotaxime and from three institutions for trimethoprim-sulfamethoxazole. In each instance, the hospital-specific proportion of *S. pneumoniae* isolates susceptible to cefotaxime or trimethoprim-sulfamethoxazole did not differ significantly by surveillance method. We were able to directly compare *S. pneumoniae* erythromycin susceptibility by antibiogram and active surveillance at six hospitals. The proportions of erythromycin-susceptible *S. pneumoniae* isolates determined by each surveillance method were statistically comparable in five of the six hospitals (p >0.05). One hospital (C) had a significantly higher proportion (p = 0.01) of erythromycin-susceptible isolates determined by active surveillance (97% [95% CI = 88% to 100%]) than reported by the corresponding antibiogram (84% [95% CI = 79% to 88%]).

### Cost Comparison

The antibiogram survey required 20 hours of health department staff time, for a total cost of $700: $650 for personnel expenses and $50 for miscellaneous support expenses. The active surveillance method required 570 hours of staff time and cost $52,000: $40,000 for direct and indirect personnel expenses and $12,000 for laboratory costs.

### Conclusions

Accurate, community-specific drug-resistant *S. pneumoniae* data are important for several reasons. First, most outpatient illnesses caused

Table 2. *Streptococcus pneumoniae* susceptibility to cefotaxime, trimethoprim-sulfamethoxazole, and erythromycin, by two surveillance methods, Portland, Oregon, 1996

| | Cefotaxime | | | | | | Trimethoprim-Sulfamethoxazole | | | | | | Erythromycin | | | | | |
|---|---|---|---|---|---|---|---|---|---|---|---|---|---|---|---|---|---|---|
| | Active surveillance | | | Antibiograms | | | Active surveillance | | | Antiobiograms | | | Active surveillance | | | Antiobiograms | | |
| | No. iso-lates | Susceptible | | No. iso-lates | Susceptible | | No. iso-lates | Susceptible | | No. iso-lates | Susceptible | | No. iso-lates | Susceptible | | No. iso-lates | Susceptible | |
| Hos-pital | | N | % | | N | % | | N | % | | N | % | | N | % | | N | % |
| A | [a] | | | [b] | | | [a] | | | [b] | | | [a] | | | [b] | | |
| B | 34 | 32 | 94 | 10 | 8 | 80 | 34 | 26 | 76 | [b] | | | 34 | 33 | 97 | 134 | 126 | 94 |
| C | 59 | 54 | 92 | 274 | 255 | 93 | 59 | 49 | 83 | 274 | 230 | 84 | 59 | 57 | 97 | 274 | 230 | 84 |
| D | 6 | 4 | 67 | 120 | 112 | 93 | 6 | 3 | 50 | | | | 6 | 5 | 83 | 120 | 88 | 73 |
| E | 12 | 12 | 100 | [b] | | | 12 | 9 | 75 | 16 | 13 | 81 | 12 | 12 | 100 | 42 | 39 | 93 |
| F | [a] | | | 61 | 59 | 97 | [a] | | | [b] | | | [a] | | | 61 | 56 | 92 |
| G | 33 | 31 | 94 | 110 | 105 | 95 | 33 | 27 | 82 | [b] | | | 33 | 30 | 91 | 110 | 100 | 91 |
| H | 11 | 11 | 100 | - | | | 11 | 10 | 91 | [b] | | | 11 | 11 | 100 | [b] | | |
| I | 8 | 7 | 88 | - | | | 8 | 6 | 75 | [b] | | | 8 | 8 | 100 | [b] | | |
| J | 7 | 6 | 86 | - | | | 7 | 5 | 71 | 10 | 8 | 80 | 7 | 6 | 86 | 10 | 10 | 100 |
| K | 6 | 6 | 100 | - | | | 6 | 4 | 67 | [b] | | | 6 | 5 | 83 | [b] | | |
| L | 2 | 2 | 100 | - | | | 2 | 2 | 100 | [b] | | | 2 | 2 | 100 | [b] | | |
| Total | 178 | 165 | 93 | 575 | 539 | 94 | 178 | 141 | 79 | 300 | 251 | 84 | 178 | 169 | 95 | 751 | 649 | 86 |

[a]Isolates submitted to the Oregon Public Health Laboratory.
[b]Antibiogram data not available from hospital.

by *S. pneumoniae* are treated empirically, without identification of the organism. Community-specific data may be a valuable determinant of empiric therapy for these infections and of initial empiric therapy for invasive disease. Second, communities with a high percentage of drug-resistant *S. pneumoniae* may benefit from efforts to reduce inappropriate antibiotic prescriptions. Increased drug-resistant *S. pneumoniae* carriage is directly related to antibiotic therapy, and reduced antimicrobial use in the community can decrease rates of antimicrobial resistance (13-16). Finally, clinicians in areas with a low percentage of drug-resistant *S. pneumoniae* and minimal penicillin resistance might gain confidence in treating presumptive outpatient infections with empiric penicillin therapy, thereby reducing the risk for multidrug resistance.

Despite the clinical and public health importance of drug-resistant *S. pneumoniae* surveillance, community-specific surveillance data are not uniformly available. A 1996 study determined that 54% of states either conducted or were planning to implement surveillance for drug-resistant *S. pneumoniae* by June 1997 (17). Our study supports the usefulness of *S. pneumoniae* antibiogram data, commonly available at many hospitals, in estimating the community-specific proportion of penicillin-susceptible *S. pneumoniae*. In no instance did hospital-specific penicillin susceptibility estimates from the two methods differ statistically. More importantly, the overall Portland penicillin susceptibility proportions determined by the active surveillance and antibiogram methods were statistically comparable.

Antibiogram data also hold promise for estimating *S. pneumoniae* susceptibilities to other antimicrobial drugs. The hospital-specific proportion of *S. pneumoniae* isolates susceptible to cefotaxime and trimethoprim-sulfamethoxazole did not differ for the two methods in hospitals where comparisons were possible. The erythromycin susceptibility proportions by antibiogram and active surveillance were statistically comparable at each of the hospitals for which erythromycin data were available, except for hospital C. The reason for this discordance is not clear but may be influenced by statistical chance.

Time and financial requirements for the antibiogram method were minimal and probably within reach of many local health departments. Laboratory effort was limited to mailing a current antibiogram to the health department. However, the antibiogram method can only estimate the proportion of drug-resistant *S. pneumoniae* in a community. The active surveillance system collects patient-specific (e.g., risk factors, demographics) and infection-specific information, permits serotyping and molecular analysis of isolates, provides data on the actual *S. pneumoniae* disease effect in the population, permits evaluation of targeted vaccination campaigns and antimicrobial guideline efforts, provides specific MIC data for a range of antimicrobial agents, and allows for validation of alternative surveillance methods.

Prior surveillance studies have documented equal or greater proportions of penicillin-resistant isolates collected from nonsterile sites than from sterile sites (18,19). Our active surveillance system captures only isolates from sterile sites collected from invasive *S. pneumoniae* disease. Most isolates in the antibiograms were from noninvasive diseases and nonsterile sites. Our study showed no statistical difference between the proportion of penicillin-susceptible *S. pneumoniae* determined by either method and therefore no difference between the penicillin-susceptible proportion of invasive and noninvasive isolates. Ninety-six percent of our active surveillance isolates were from outpatients or inpatients hospitalized less than 48 hours and are unlikely to represent nosocomial infections. These data support the traditional epidemiologic characterization of *S. pneumoniae* as a community rather than nosocomially acquired organism.

Several potential limitations deserve comment. The active surveillance system had a case-isolate recovery rate of 67%. The current performance indicator for the active surveillance system, instituted in 1998, is a case-isolate recovery rate of 85% (A. Schuchat, pers. comm.) We were unable to characterize the susceptibilities of the missing isolates, which may have biased our active surveillance results. Antibiograms were tabulated from all isolates submitted to a particular hospital laboratory. Multiple isolates from a single patient may have disproportionately influenced these results. Unlike the active surveillance system, in which chart reviews excluded nonresidents, antibiogram data may have included isolates from patients who were not Portland-area residents and should not have been included in Portland *S. pneumoniae* antimicrobial-susceptibility results. We were unable to estimate the number of duplicate isolates or non-Portland-area residents in our antibiogram data.

This study suggests that antibiogram data already available in hospitals may be useful in estimating the community-specific proportion of drug-resistant *S. pneumoniae*. We recommend further validation of these results at sites where active surveillance and antibiogram data can be directedly compared. The most effective use of antibiogram drug-resistant *S. pneumoniae* surveillance may require that hospitals routinely and consistently perform *S. pneumoniae* susceptibility testing to multiple antimicrobial drugs. Communities considering this surveillance method may need to work with local hospitals to develop a cost-effective susceptibility testing regimen. Although yielding less information than active surveillance, antibiogram surveillance might be most useful in communities where hospital antibiogram data are available but more intensive surveillance is limited by a lack of financial or personnel resources.

### Acknowledgments

We thank the laboratory directors, managers, and staff at the Oregon State Public Health Laboratory and the Portland Tri-County hospital microbiology laboratories for their work in this study and Drs. Anne Schuchat, Thomas Van Gilder, and Andrew Pelletier for manuscript review.

This study was funded, in part, by a cooperative agreement between the Oregon Health Division and the Centers for Disease Control and Prevention's Emerging Infections Program.

At the time this study was performed, Dr. Chin was an Epidemic Intelligence Service Officer with the Centers for Disease Control and Prevention, assigned to the Oregon Health Division. He currently practices emergency medicine in Sharon, Connecticut.

### References

1. Hansman D, Bullen MM. A resistant pneumococcus. Lancet 1967;July 29, 1967:264-5.
2. Breiman RF, Butler JC, Tenover FC, Elliott JA, Facklam RR. Emergence of drug resistant-pneumococcal infections in the United States. JAMA 1994;271:1831-5.
3. Friedland IR, McCracken GH. Management of infections caused by antibiotic-resistant *Streptococcus pneumoniae*. N Engl J Med 1994;331:377-82.
4. Butler JC, Hofmann J, Cetron MS, Elliott JA, Facklam RR, Breiman RF. The continued emergence of drug-resistant *Streptococcus pneumoniae* in the United States: an update from the Centers for Disease Control and Prevention's pneumococcal sentinel surveillance system. J Infect Dis 1996;174:986-93.
5. Doern GV, Pfaller MA, Kugler K, Freeman J, Jones RN. Prevalence of antimicrobial resistance among respiratory tract isolates of *Streptococcus pneumoniae* in North America: 1997 results from the SENTRY antimicrobial surveillance program. Clin Infect Dis 1998;27:764-70.
6. Centers for Disease Control and Prevention. Drug-resistant *Streptococcus pneumoniae*—Kentucky and Tennessee, 1993. MMWR Morb Mortal Wkly Rep 1994;43:23-6.
7. Friedland IR, Shelton S, Paris M, Rinderknecht S, Ehrett S, Krisher K, et al. Dilemmas in diagnosis and management of cephalosporin-resistant *Streptococcus pneumoniae* meningitis. Pediatr Infect Dis J 1993;12:196-200.
8. Kleiman MB, Weinberg GA, Reynolds JK, Allen SD. Meningitis with beta-lactam-resistant *Streptococcus pneumoniae*: the need for early repeat lumbar puncture. Pediatr Infect Dis J 1993;12:782-4.
9. Frick PA, Black DJ, Duchin JS, Deliganis S, McKee WM, Fritsche TR. Prevalence of antimicrobial drug-resistant *Streptococcus pneumoniae* in Washington State. West J Med 1998;169:364-9.
10. Jernigan DB, Cetron MS, Breiman RF. Minimizing the impact of drug-resistant *Streptococcus pneumoniae* (DRSP): a strategy from the DRSP working group. JAMA 1996;275:206-9.
11. Centers for Disease Control and Prevention. Defining the public health impact of drug-resistant *Streptococcus pneumoniae*: report of a working group. MMWR Morb Mortal Wkly Rep 1996;45(RR-1):1-20.
12. National Committee for Clinical Laboratory Standards. Methods for dilution antimicrobial susceptibility tests for bacteria that grow aerobically. 4th ed. Approved standard. NCCLS [document M7-A4]. Vol 17. Wayne (PA): The Committee; 1997.
13. Reichler MR, Allphin AA, Breiman RF, Schreiber JR, Arnold JE, McDougal LK, et al. The spread of multiply resistant *Streptococcus pneumoniae* at a day care center in Ohio. J Infect Dis 1992;166:1346-53.
14. Pallares R, Gudiol F, Linares J, Ariza J, Rufi G, Murgui L, et al. Risk factors and response to antibiotic therapy in adults with bacteremic pneumonia caused by penicillin-resistant pneumococci. N Engl J Med 1987;317:18-22.
15. Leach AJ, Shelby-James TM, Mayo M, Gratten M, Laming AC, Currie BJ, et al. A prospective study of the impact of community-based azithromycin treatment of trachoma on carriage and resistance of *Streptococcus pneumoniae*. Clin Infect Dis 1997;24:356-62.
16. Seppala H, Klaukka T, Vuopio-Varkila J, Muotiala A, Helenius H, Lager K, et al. The effect of changes in the consumption of macrolide antibiotics on erythromycin resistance in group A streptococci in Finland. N Engl J Med 1997;337:441-6.
17. Centers for Disease Control and Prevention. Assessment of national reporting of drug-resistant *Streptococcus pneumoniae*—United States, 1995-1996. MMWR Morb Mortal Wkly Rep 1996;45:947-50.
18. Heffernan R, Henning K, Labowitz A, Hjelte A, Layton M. Laboratory survey of drug-resistant *Streptococcus pneumoniae* in New York City, 1993-1995. Emerg Infect Dis 1998;4:113-6.
19. Kellner J, McGeer A, Cetron M, Low D, Butler J, Matlow A, et al. The use of *Streptococcus pneumoniae* nasopharyngeal isolates from healthy children to predict features of invasive disease. Pediatr Infect Dis J 1998;17:279-86.
